# Feeding patterns and BMI trajectories during infancy: a multi-ethnic, prospective birth cohort

**DOI:** 10.1186/s12887-020-02456-4

**Published:** 2021-01-13

**Authors:** Outi Sirkka, Michel H. Hof, Tanja Vrijkotte, Marieke Abrahamse-Berkeveld, Jutka Halberstadt, Jacob C. Seidell, Margreet R. Olthof

**Affiliations:** 1 Department of Health Sciences, Faculty of Science, Vrije Universiteit Amsterdam, Amsterdam Public Health Research Institute, De Boelelaan 1085, 1081 HV Amsterdam, the Netherlands; 2 Danone Nutricia Research, Utrecht, the Netherlands; 3 Department of Clinical Epidemiology, Biostatistics and Bioinformatics, Amsterdam Public Health Research institute, Amsterdam UMC, University of Amsterdam, Amsterdam, The Netherlands; 4 Department of Public and Occupational Health, Amsterdam Public Health Research Institute, Amsterdam UMC, University of Amsterdam, Amsterdam, the Netherlands

**Keywords:** Infant feeding, Breastfeeding, Complementary feeding, BMI trajectories, Overweight

## Abstract

**Background:**

Milk feeding type (exclusive breastfeeding [EBF], formula feeding or mixed feeding) and timing of complementary feeding (CF) have been associated with infant growth. However, studies evaluating their combined role, and the role of ethnicity, are scarce. We examined associations of feeding patterns (milk feeding type combined with timing of CF) with infant body mass index (BMI) trajectories and potential ethnic-specific associations.

**Methods:**

Infant feeding and BMI data during the 1st year of life from 3524 children (Dutch *n* = 2880, Moroccan *n* = 404 and Turkish *n* = 240) from the Amsterdam Born Children and their Development (ABCD) cohort were used. Six feeding patterns were defined: EBF/earlyCF, EBF/lateCF (reference), formula/earlyCF, formula/lateCF, mixed/earlyCF and mixed/lateCF. A covariate adjusted latent class mixed model was applied to simultaneously model BMI trajectories and associations with feeding patterns. Potential ethnic differences in the associations were studied in a separate model where interactions between ethnicity and feeding patterns were included.

**Results:**

Four distinct BMI trajectories (low, mid-low, mid-high and high) were identified. Feeding pattern of formula/earlyCF was associated with lower odds for low (OR: 0.43; 95% CI: 0.25, 0.76) or mid-high (0.28; 0.16, 0.51) (ref: high) trajectory compared with EBF/lateCF pattern (ref). An ethnic-specific model revealed that among Dutch infants, formula/earlyCF pattern was associated with lower odds for low trajectory (0.46; 0.24, 0.87), whereas among Turkish/Moroccan infants almost all feeding patterns were associated with lower odds for the low trajectory (ref: high).

**Conclusion:**

Infant feeding patterns are associated with early BMI trajectories with specific ethnic differences. Future studies should take the role of ethnicity into account in the associations between infant feeding and growth.

**Supplementary Information:**

The online version contains supplementary material available at 10.1186/s12887-020-02456-4.

## Background

Childhood overweight and obesity may track into adulthood and are associated with adverse health outcomes from childhood [1, 2]. Rapid growth, i.e. excess weight gain or excess increase in BMI (kg/m^2^), during 1st year of life is associated with increased risk of later life overweight [3–5]. Infant feeding is suggested as one of the most important modifiable factors associated with early growth trajectories and later overweight and obesity [6–8].

Milk feeding type, e.g. exclusive breastfeeding (EBF), formula feeding or a combination thereof (mixed feeding) has been associated with infant growth outcomes [9–11]. In general, (exclusive) breastfeeding has been associated with slower weight and length gain during infancy [12], lower BMI and lower risk of childhood overweight [13] compared with formula feeding. Yet some studies have reported no association between breastfeeding and growth outcomes during infancy [14], childhood [15, 16] or adulthood [17, 18]. Methodological differences across studies, i.e. adjustment for confounders or definitions of breastfeeding might explain these apparent discrepancies [13]. Several studies combined mixed-fed and EBF infants into one breastfed group [19, 20] whereas, in other studies, mixed-fed infants were either considered as formula-fed [21] or excluded from the analysis [9]. In addition to milk feeding type, some evidence suggests that the timing of complementary feeding (CF) may influence body weight and BMI during childhood, yet evidence is mixed [22]. Later CF has been associated with lower prevalence of childhood and adult overweight [16, 17]. Other studies suggested reverse causality [23] or no associations [24–26] between timing of CF and infant weight gain or childhood overweight.

Most previous studies investigating the associations of milk feeding type or timing of CF have mutually adjusted for these factors to evaluate their independent effects on later growth outcomes. However, some studies reported an interaction between these factors [27–29]. Early CF in formula- or mixed-fed infants has been associated with increased infant weight gain [27] or childhood overweight [28]. On the contrary, one study reported that late CF introduction among EBF infants was associated with an increased prevalence of overweight [29]. Therefore, the combination of different milk feeding types with timing of CF should be further investigated.

Considerable ethnic differences exist in infant feeding practices and childhood overweight prevalence [30–32]. In the Netherlands, mothers of Turkish or Moroccan ethnicity are reported to provide longer duration of EBF or mixed feeding than mothers of Dutch ethnicity [33, 34]. However, children from Turkish or Moroccan ethnicities have a higher infancy weight gain and childhood overweight prevalence compared to children of Dutch ethnicity [32, 33, 35]. Hence, it is of interest to improve our understanding of potential ethnic differences in the association between infant feeding patterns and growth.

Our main objectives were: (i) examine associations of feeding patterns (i.e. milk feeding type during the first 3 months of life combined with timing of CF) with distinct infant BMI trajectories and (ii) determine potential ethnic differences in these associations. Additionally, we examined overweight prevalence at 5–6 years among the identified BMI trajectories.

## Methods

### Subjects

**Fig. 1:**
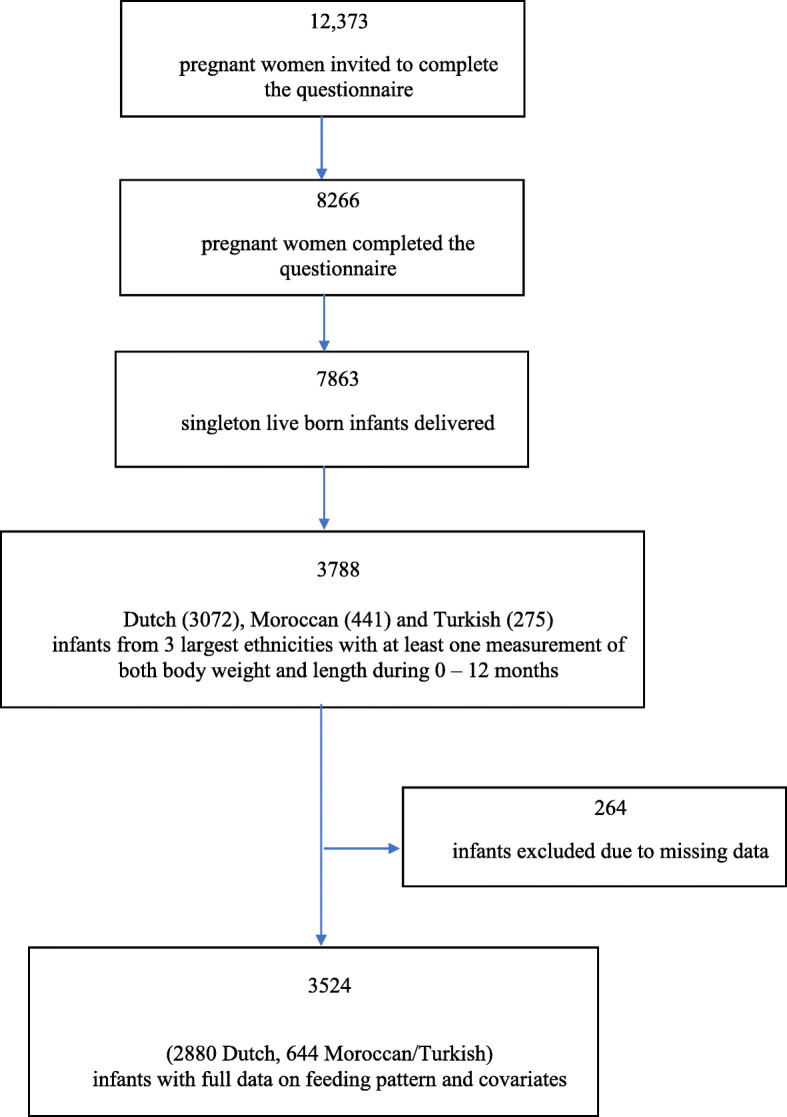
A flowchart of the selection of analyzed study population

Data were obtained from the Amsterdam Born Children and their Development (ABCD) study, a large prospective birth cohort in Amsterdam, the Netherlands [36]. Between January 2003 and March 2004, all pregnant women (*n* = 12,373) living in Amsterdam were invited to participate in this study by filling out a pregnancy questionnaire. Of these women, 8266 women completed the questionnaire during their 12–14th week of pregnancy and 7863 gave birth to a live singleton infant. For the purpose of the current study, infants from the three largest ethnic groups with at least one measurement of both body weight and length during the first year were included; Dutch (*n* = 2998), Moroccan (*n* = 437) and Turkish (*n* = 270). Ethnicity of the mother and her infant was defined as the country of birth of the mother or maternal grandmother to include first and second generation immigrants [37]. Moroccan and Turkish ethnicities were of particular interest due to relatively high prevalence of overweight from early childhood onwards [35]. These ethnic groups were combined for the analysis because of the low number of participants in both groups and previously reported similarities in infant feeding and growth of the infants [33, 34]. Children with missing values on feeding pattern (*n* = 97) or at least one of the following covariates: maternal educational level (*n* = 17), maternal pre-pregnancy BMI (*n* = 10), maternal smoking during pregnancy (*n* = 137), gestational age (*n* = 3), birth weight (*n* = 10) were excluded. The final study sample consisted of 3524 children (Fig. 1). The ABCD study was approved by the Central Committee on Research Involving Human Subjects, the Medical Ethical Examining Committees of all Amsterdam Hospitals and the Municipal Privacy Protection Committee of Amsterdam and was developed in accordance with the Declaration of Helsinki. All participants provided written informed consent.

### Measures

#### Infant feeding pattern

Information about infant feeding (EBF duration and the child’s age when receiving formula and CF) were collected by using a questionnaire, administered at the Youth Health Care (YHC) routine visits during the first year. These data were recorded in following categories: none, < 1 month, 1–2.9 months, 3–5.9 months and > =6 months. More detailed description of the data collection procedure has been previously described [33]. EBF was defined as providing only breastmilk, no other solids/fluids except water. CF was defined as any solids or fluids other than breastmilk, infant formula or water.

From the available data, infant feeding patterns were defined based on the milk feeding type provided combined with the timing of CF introduction. We initially aimed to investigate feeding pattern during 0–6 months according to the World Health Organization (WHO) recommendations to give EBF until 6 months [38], however, the majority of women in the Netherlands discontinue EBF after 3 months [39]. One of the main reasons to discontinue EBF after 3 months is that it is common for women in the Netherlands to return to work after 3 months from the delivery. Prevalence of EBF ≥6 months in our study was only around 18%, close to the Dutch national estimates reported at the time of the study [40]. Therefore, for the milk feeding type, a period of 0–3 months was chosen and sensitivity analyses using the milk feeding type during 0–6 months were carried out. Three milk feeding categories during 0–3 months were established: 1) EBF, defined as exclusive breastfeeding from birth onwards; 2) formula feeding, defined as introducing full formula feeding within the first month after birth and 3) mixed feeding, as any other milk feeding combination which was not described above. Timing of CF was dichotomized as” early” (< 6 months) or” late” (≥6 months) according to the WHO [38]. Although other recommendations exist to introduce CF between 4 and 6 months [41], the numbers for the feeding pattern combinations were not sufficient for the ethnic-specific analyses when using this categorization. In our dataset the vast majority (80% of the mothers) introduced CF between 4 and 6 months, only around 5% before 4 months and 15% after 6 months.

By combining the three categories of milk feeding type and the two categories of CF timing, infants were classified into six feeding patterns: 1) EBF with late CF (EBF/lateCF); 2) EBF with early CF (EBF/earlyCF); 3) formula feeding with late CF (formula/lateCF); 4) formula feeding with early CF (formula/earlyCF); 5) mixed feeding with late CF (mixed/lateCF); and 6) mixed feeding with early CF (mixed/earlyCF). For the analyses, EBF/lateCF feeding pattern was chosen as the reference since this most closely reflects the WHO recommendations [38].

#### BMI measurements during infancy and at 5–6 years

Data on weight and length (BMI) during the first year were collected during the YHC routine visits where children are invited to at 1, 2, 3, 4, 6, 7.5, 9, and 11 months of age. These data were obtained from the YHC registry. During these visits, height was measured to the nearest millimeter with a Leicester portable height measure (Seca, Hamburg, Germany). Weight was measured to the nearest 100 g with a calibrated Marsden M-4102 scale (Oxfordshire, UK) [36]. From these data, BMI was calculated as weight in kilograms divided by the square of height in meters. For deriving BMI trajectories, non-standardized BMI values were used. At 5–6 years of age, data on child’s weight and height (for BMI) were obtained from the YHC registry (*n* = 1235) or the ABCD health examinations (*n* = 1868). The examinations were conducted by trained research assistants according to a standard protocol [33]. Age- and sex- specific BMI standard deviation (SD) scores were derived according to the WHO growth standards [42] using the Growth Analyzer Software, version 4.0 (Growth Analyzer BV). Overweight (including obesity) was defined as > + 1SD above the median of the WHO growth standards.

#### Covariates

Data on predefined maternal covariates; pre-pregnancy BMI, educational level, parity and smoking (during pregnancy), were obtained through the pregnancy questionnaire and were self-reported by the mother. Pre-pregnancy BMI was dichotomized as normal weight (BMI < 25 kg/m^2^) or overweight (including obesity) (≥25 kg/m^2^). Educational level was defined as years of education after primary school and categorized as either low: 0–5 years of education; medium: 6–10 years or high: > 10 years [43]. Smoking (no/yes) and parity (primipara/multipara) were dichotomized. Infancy covariates included sex, birth weight (in kg) and gestational age which was dichotomized (term: ≥37 weeks and preterm: < 37 weeks of gestational age). These data were obtained from the YHC registry. Height was not measured at birth and therefore BMI at birth could not be calculated. Trajectories were based on the first available BMI data (usually obtained in the first months after birth) and onwards.

### Statistical analysis

A latent class mixed effect model (LCMM) was fitted to the BMI data during infancy [44]. With the LCMM model, we simultaneously estimated *k* BMI trajectories (i.e. latent classes) and the probability that an infant followed a particular BMI trajectory. The class membership of an infant *i* was defined by the unobserved discrete variable *C*_*i*_, where *C*_*i*_ = *g* if infant *i* followed BMI trajectory *g* = 1,.., *k*. Since it was not known before performing the analysis how many trajectories were present in the data, the number of BMI trajectories *k* was determined based on the Bayesian Information Criterion. The LCMM consists of two submodels; the linear mixed effect submodel describing each trajectory and the multinomial logistic regression submodel describing the associations between feeding patterns and the infant’s probability to follow a particular trajectory.

#### Linear mixed effect submodel

The linear mixed effect submodel for BMI trajectory *g* =1, …, *k* was parameterized as follows. For the fixed effects, natural cubic spline functions with five degrees of freedom were used. The inner knots of the spline functions were placed at the corresponding percentiles of the data. Additionally, a trajectory specific random intercept and a trajectory specific random slope were used to capture the correlation between the BMI measurements from each infant. This led to the following submodel:

$$ {\left.{Y}_{ij}\right|}_{C_i=g}=f\left({t}_{ij};{\boldsymbol{\beta}}_g\right)+{b}_{ig,1}+{t}_{ij}{b}_{ig,2}+{\epsilon}_{ij} $$

where *y*_*ij*_ is the *j*^*th*^ BMI measurement of infant *i* obtained at age *t*_*ij*_ and ***f***(*t*_*ij*_, ***β***_*g*_) is the natural spline function describing the *g*^*th*^ BMI trajectory parametrized by the vector ***β***_***g***_. In addition, (*b*_*ig*, 1_, *b*_*ig*, 2_) are the random slope and intercept for the *g*^*th*^ BMI trajectory and *ϵ*_*ij*_ is a residual term. In our model, the random effects (*b*_*ig*, 1_, *b*_*ig*, 2_) were assumed to follow a bivariate normal distribution with means zero and an unstructured covariance matrix Σ. The residual *ϵ*_*ij*_ was assumed to follow a normal distribution with mean zero and variance parameter σ.

#### Multinomial logistic regression submodel

The multinomial logistic regression submodel described the probability that infant *i* followed trajectory *g*. For the two main objectives of this study, two separate LCMM analyses were performed. First, to examine whether feeding patterns were associated with distinct infant trajectories, an additive relation was assumed for all covariates. In this model, we assumed that the effects of the six feeding patterns were the same in Dutch and non-Dutch (i.e. Turkish and Moroccan) infants. With this LCMM, referred to as model 1, the probability that infant *i* followed trajectory *g* was given by

$$ \mathit{\Pr}\left({C}_i=g{\left|\boldsymbol{\mathsf{x}}\right.}_i\right)=\frac{\exp \left({\gamma}_{g0}+{\mathit{\mathsf{x}}}_{i1}{\gamma}_{g1}+{\mathit{\mathsf{x}}}_{i2}{\gamma}_{g2}+{\mathit{\mathsf{x}}}_{i3}{\gamma}_{g3}+\cdots +{\mathit{\mathsf{x}}}_{ip}{\gamma}_{gp}\right)}{\sum_{j=1}^k\exp \left({\gamma}_{j0}+{\mathit{\mathsf{x}}}_{i1}{\gamma}_{j1}+{\mathit{\mathsf{x}}}_{i2}{\gamma}_{j2}+{\mathit{\mathsf{x}}}_{i3}{\gamma}_{j3}+\cdots +{\mathit{\mathsf{x}}}_{ip}{\gamma}_{jp}\right)}, $$

Second, to explore potential ethnic differences in the associations of feeding patterns with the trajectories, we combined the feeding patterns and the ethnicity variable into a dummy variable with pre-determined 12 unique values (i.e. 6 feeding patterns for Dutch and 6 feeding patterns for Turkish/Moroccan). In this model, referred to as model 2, the feeding patterns were allowed to have different effects in the Dutch and Turkish/Moroccan infants. In both models, the reference group were Dutch infants with EBF/lateCF pattern. All analyses were conducted using the statistical program R 3.5.2 with package lcmm [44].

### Overweight at 5–6 years of age

Based on the fitted LCMM, we calculated the posterior probability of following a certain trajectory for each child. These probabilities were used as weighing of a child’s contribution to each trajectory. For example, a child could contribute to 20, 45 and 35% respectively, to the first, second, and third trajectory. Using the information on child’s BMI at 5–6 years, we then derived the percentage of children with overweight (or obesity) within each trajectory, according to the child’s weighted contribution to each trajectory.

## Results

### Participants

Table 1 shows the characteristics of the full study population as well as based on ethnicity or the pre-defined feeding patterns. Overall, the most common pattern was EBF/lateCF, including 30% of the participants. Among the Turkish/Moroccan mothers, mixed feeding or EBF/late CF introduction were the 3 largest groups. For Dutch mothers, most apparent feeding group was EBF/late CF with one third of mothers.

**Table 1:** Characteristics of the study population by ethnicity and feeding pattern^a^

		Ethnicity		Feeding pattern					
		Dutch	Turkish/ Moroccan	EBF/ early CF	EBF/ late CF	Mixed/ early CF	Mixed/ late CF	Formula/ early CF	Formula/ late CF
	*All (3524)*			16.0 (562)	31.2 (1101)	16.2 (573)	16.9 (597)	11.5 (405)	8.1 (286)
*Maternal characteristics*									
Ethnicity^b^									
*Dutch*	81.7 (2880)			16.5 (475)	32.7 (943)	14.8 (428)	15.1 (436)	12.1 (348)	8.7 (250)
*Turkish / Moroccan*	18.3 (644)			13.5 (87)	24.5 (158)	22.5 (145)	25.0 (161)	8.8 (57)	5.6 (36)
Education									
*Low*	15.7 (554)	6.9 (199)	55.1 (355)	12.3 (69)	11.0 (121)	17.1 (98)	22.1 (132)	21.0 (85)	17.1 (49)
*Medium*	34.8 (1226)	33.9 (976)	38.8 (250)	33.3 (187)	30.0 (330)	39.1 (224)	33.8 (202)	41.0 (166)	40.9 (117)
*High*	49.5 (1744)	59.2 (1705)	6.1 (39)	54.4 (306)	59.1 (650)	43.8 (251)	44.1 (263)	38.0 (154)	42.0 (120)
Pre-pregnancy BMI									
*Overweight*	21.6 (761)	17.0 (491)	41.9 (270)	19.4 (109)	18.7 (206)	20.2 (116)	24.8 (148)	29.9 (121)	21.3 (61)
Parity									
*Primiparous*	57.0 (2010)	59.5 (1713)	46.1 (297)	52.3 (294)	52.6 (579)	60.9 (349)	58.1 (347)	60.0 (243)	69.2 (198)
Smoking									
*Yes*	6.6 (232)	6.6 (191)	6.4 (41)	5.2 (29)	4.9 (54)	7.2 (41)	5.9 (35)	10.4 (42)	10.8 (31)
*Child characteristics*									
Sex									
*Boy*	50.3 (1773)	49.5 1.4(115)	53.8 (347)	49.1 (276)	49.5 (545)	51.7 (296)	50.6 (302)	51.6 (209)	50.7 (145)
Preterm birth									
*Yes*	4.0 (140)	4.1 (118)	3.4 (22)	0.9 (5)	3.2 (35)	3.8 (22)	4.5 (27)	6.2 (25)	9.1 (26)
Birth weight (*kg*)									
*mean*, *SD*	3.51 (0.53)	3.52 (0.54)	3.44 (0.51)	3.58 (0.50)	3.54 (0.50)	3.49 (0.50)	3.45 (0.55)	3.49 (0.59)	3.43 (0.63)

^a^Values are % (N), unless otherwise indicated. ^b^The percentages for the different feeding patterns are within the Dutch or within the Turkish ethnicity

### Classification of BMI trajectories

**Fig. 2:**
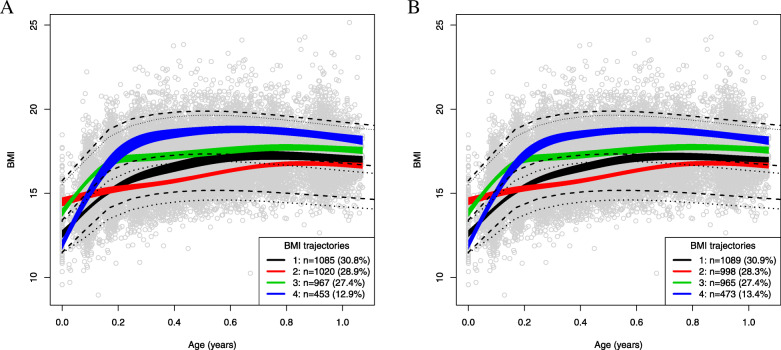
**a**. BMI trajectories identified by the LCMM, model 1. **b**. BMI trajectories identified by the LCMM, model 2

Four BMI trajectories during the first year of life were identified in models 1 and 2 (Fig. 2). Both models resulted in similar trajectories with respect to the shape of the trajectories and the number of infants assigned to each trajectory. The trajectories were classified as low, mid-low, mid-high and high according to the WHO BMI reference percentiles [45]. The most prevalent trajectory, named as “low”, described a relatively stable BMI pattern with values below the median of the WHO growth standards through the 1st year of life. Trajectories “mid-low” and “mid-high” showed an ascending pattern in the BMI values close to or slightly above the median of the WHO growth standard. The trajectory “high” showed a rapid BMI increase during the first 2 months of life and remained at a substantially high BMI value throughout the year, well-above the median of the WHO growth standard.

### Associations of feeding patterns with BMI trajectories

Table 2 presents the results of model 1 describing the covariate adjusted associations between feeding patterns and BMI trajectories. Compared with the reference feeding pattern (EBF/lateCF), infants with a pattern of formula/earlyCF had lower odds of belonging to the low rather than to the high trajectory. Furthermore, formula/earlyCF and mixed/earlyCF were associated with lower odds of belonging to mid-high trajectory compared with the high trajectory.

**Table 2:** Associations of infant feeding patterns with BMI trajectories^a, b, c^

	BMI trajectory					
	“low”		“mid-low”		“mid-high”	
	OR (95% CI)	*p*-value	OR (95% CI)	*p*-value	OR (95% CI)	*p*-value
Feeding patterns						
EBF/ early CF	0.86 (0.52, 1.42)	0.56	0.94 (0.50, 1.79)	0.85	0.75 (0.44, 1.28)	0.29
EBF/ late CF (reference)	–		–		–	
Mixed/ early CF	0.63 (0.35, 1.15)	0.12	1.71 (0.96, 3.03)	0.14	0.50 (0.29, 0.87)	0.01
Mixed/ late CF	0.62 (0.33, 1.17)	0.64	1.55 (0.80, 3.00)	0.13	0.47 (0.25, 0.89)	0.19
Formula/ early CF	0.43 (0.25, 0.76)	<0.01	0.76 (0.40, 1.44)	0.40	0.28 (0.16, 0.51)	<0.01
Formula/ late CF	0.63 (0.27, 1.48)	0.29	1.71 (0.73, 4.00)	0.22	0.50 (0.24, 1.07)	0.07

^a^Values are OR based on LCMM. ^b^Reference BMI trajectory “high”. ^c^Adjusted for ethnicity, educational level, pre-pregnancy BMI, parity, smoking, sex, preterm birth, birth weight

### Ethnic-specific associations of infant feeding patterns with BMI trajectories

The results from model 2 including the ethnic-specific associations (Table 3) revealed that compared with the reference group (EBF/lateCF, Dutch infants), formula/earlyCF pattern of Dutch infants was associated with lower odds for the low compared with the high trajectory. Interestingly, compared with the reference group, all feeding patterns of Turkish/Moroccan infants indicated lower odds of being in the low rather than high trajectory.

**Table 3:** Ethnic-specific associations of infant feeding patterns with BMI trajectories (model 2) (Dutch, Turkish/Moroccan)^a, b, c^

	BMI trajectory					
	“low”		“mid-low”		“mid-high”	
	OR (95% CI)	p-value	OR (95% CI)	*p*-value	OR (95% CI)	*p*-value
Feeding pattern						
Dutch						
EBF/ early CF	1.09 (0.59, 2.01)	0.79	1.14 (0.54, 2.41)	0.73	0.90 (0.46, 1.75)	0.75
EBF/ late CF (reference)	–		–		–	
Mixed/ early CF	0.62 (0.32, 1.22)	0.28	1.46 (0.74, 2.85)	0.12	0.54 (0.27, 1.08)	0.05
Mixed/ late CF	0.69 (0.32, 1.49)	0.70	1.72 (0.75, 3.95)	0.22	0.50 (0.21, 1.19)	0.35
Formula/ early CF	0.46 (0.24, 0.87)	0.02	0.79 (0.39, 1.57)	0.50	0.30 (0.15, 0.61)	<0.01
Formula/ late CF	0.62 (0.26, 1.49)	0.29	1.46 (0.58, 3.63)	0.42	0.54 (0.23, 1.24)	0.15
Turkish/Moroccan						
EBF/ early CF	0.14 (0.05, 0.42)	<0.01	0.15 (0.03, 0.68)	0.01	0.50 (0.21, 1.20)	0.12
EBF/ late CF	0.43 (0.20, 0.91)	0.03	0.32 (0.13, 0.82)	0.02	0.72 (0.33, 1.56)	0.41
Mixed/ early CF	0.14 (0.06, 0.33)	<0.01	0.39 (0.15, 1.00)	0.05	0.30 (0.14, 0.65)	<0.01
Mixed/ late CF	0.25 (0.11, 0.55)	<0.01	0.44 (0.18, 1.07)	0.07	0.41 (0.18, 0.94)	0.03
Formula/ early CF	0.11 (0.03, 0.39)	<0.01	0.22 (0.06, 0.80)	0.02	0.18 (0.05, 0.61)	0.01
Formula/ late CF	0.06 (0.00, 1.50)	0.09	0.92 (0.31, 2.73)	0.88	0.22 (0.04, 1.12)	0.07

^a^Values are OR based on LCMM. ^b^Reference BMI trajectory “high”. ^c^Adjusted for covariates: educational level, pre-pregnancy *BMI* parity, smoking, sex, preterm birth, birth weight

Sensitivity analyses including the milk feeding type during 0–6 months, where the reference feeding pattern (EBF/late CF) was defined as EBF ≥ 6 months/CF ≥ 6 months, showed comparable results for most feeding groups compared to our main results (Additional file 1). However, compared to the reference feeding pattern, mixed/late CF (i.e. either mixed feeding or EBF < 6 months combined with CF ≥ 6 months) was associated with higher odds for the low trajectory. Ethnic-specific model showed that among the Dutch both mixed/early CF and mixed/late CF were associated with higher odds for the low trajectory.

### Overweight at 5–6 years

The lowest percentage of overweight at 5–6 years of age was observed for the low trajectory (10.1%) whereas the highest was seen for the high trajectory (34.1%); the other groups were intermediate (Table 4). Compared with the Dutch children, percentage of Turkish/Moroccan children with overweight appeared to be higher in all trajectories.

**Table 4:** Overweight prevalence at age 5–6 years in the BMI trajectories (*n* = 2753)^a^

	% of overweight in each BMI trajectory			
	“low”	“mid-low”	“mid-high”	“high”
Total (*n* = 2753)	10.1	12.2	22.6	34.1
Dutch (*n* = 2253)	8.6	9.7	18.4	27.4
Turkish/Moroccan (*n* = 500)	23.6	26.4	38.5	45.0

^a^Overweight (including obesity): > + 1 SD above the median of the age and sex-specific WHO 2007 growth standards

## Discussion

Our results showed that specific infant feeding patterns are associated with BMI trajectories during the first year of life and that there are apparent ethnic differences in these associations. Compared with infants who received EBF during the first 3 months with CF introduction after 6 months (reference), infants who were formula-fed with early CF introduction had lower odds for being in the low rather than high trajectory. Ethnic-specific analyses revealed that Dutch infants with a feeding pattern of formula feeding and early CF had lower odds for being in the low trajectory rather than in the high. Among Turkish/Moroccan infants, all feeding patterns were associated (tended to) with lower odds for being in the low trajectory compared with the Dutch (reference) infants.

To our knowledge, three previous studies [27, 46, 47] investigated different combinations of milk feeding types or breastfeeding duration with timing of CF on growth outcomes during infancy. These studies showed inconsistent findings and used somewhat different feeding pattern definitions than our study. In line with our findings, Imai et al. [46] reported greater weight gain during infancy and higher BMI at 6 years among infants who were formula-fed and introduced to CF early (≤5 months) compared to infants receiving EBF for 5 months. However, unlike our study, they did not investigate the group of EBF infants with early CF exposure. The other two studies investigated breastfeeding duration (EBF or mixed feeding) with timing of CF. Baker et al. found that a short duration (< 5 months) of breastfeeding with early CF (4 months) was associated with an increased infant weight gain compared with long duration (> 10 months) of breastfeeding with late CF (≥4 months) [27]. Sun et al. however reported that regardless of breastfeeding duration, early CF (< 4 months) compared with late CF (≥4 months) was associated with above normal BMI at 1 year [47]. Furthermore, previous studies on feeding patterns and later overweight suggested that early (< 4 months) compared with late (≥4 months) CF was associated with childhood overweight only in formula-fed/short breastfed infants [28, 29, 48, 49]. finally, two studies suggested that also delayed CF (> 7 months) was associated with overweight in later life among children receiving EBF [29, 47]. Differences in the study populations, definitions or cut-offs of the feeding exposures are likely to explain the mixed findings. Interestingly, in our sensitivity analyses including milk feeding during 0–6 months, compared to the reference pattern, mixed feeding/lateCF was associated with higher odds for the low trajectory. These results could be partly explained by differences in some characteristics between the reference groups (EBF ≥ 6 months/lateCF vs. EBF ≥ 3months/lateCF in our main analysis). Mothers in the EBF ≥ 6 months/late CF group were less often highly educated (53.6 vs 59.1%), had more often overweight (22.2 vs 18.7%) and had Turkish/Moroccan ethnic background (17.2 vs 14.4%) compared with the reference group in our main analysis. Furthermore, the majority of mothers (51%) in the mixed/late CF group provided rather long duration of EBF (between 3 and 6 months). Due to some very small group sizes (i.e. Dutch EBF/earlyCF only 5.2%) these results should be interpreted with caution. We also cannot exclude the possibility of chance findings. Given the evidence both on the benefits of EBF ≥6 months [13, 27] as well as introducing CF between the age of 4–6 months [41], future studies assessing growth the outcomes should consider alternative categorization of feeding patterns.

In addition to the differences in feeding definitions, large differences exist in the adjustment for confounders across studies. Especially, previous studies on breastfeeding and childhood BMI showed that the associations were largely reduced after adjustment for family-based sociodemographic, or maternal/child lifestyle related factors (i.e. sedentary activities, sleep duration or dietary pattern in toddlerhood) [50, 51]. Although the findings from previous studies on early growth or overweight are controversial and comparison of studies is difficult due to different feeding definitions, evidence from our and several other studies does suggest that especially the combination of formula feeding during early infancy with early CF introduction is associated with rapid infant growth or childhood overweight.

There are several explanations for the observed associations of feeding patterns on infant BMI in our study. Formula feeding is suggested to lead to a more rapid growth during infancy compared to breastfeeding [52]. Possible mechanisms include the composition of formula milk, e.g. higher protein content compared to breastmilk as well as differences in feeding style such as feeding on schema, which may lead to lower ability to self-regulate food intake [53]. Infants who are exposed to formula feeding and early CF may have higher energy and/or protein intakes compared with infants with EBF and late CF [54]. These higher intakes from both milks and foods may consequently lead to more rapid gains in weight (and length), explaining their lower odds for being in the low BMI trajectory [55, 56]. However, we did not have information on the quantity and quality of diet during infancy which may explain the associations observed in our study [54, 57]. It is also possible that our results may reflect reverse causality rather than causal associations; infants who grow more rapidly may have higher energy needs and demand for feedings. This could lead to parents switching from breastfeeding to formula feeding and to introducing CF earlier to these children [23]. However, we have no information on reasons as to why formula was chosen or why CF was introduced.

We observed substantial ethnic differences in the associations of feeding patterns with BMI trajectories, suggesting that ethnicity may modify this association. Nevertheless, irrespective of ethnicity, infants with formula/earlyCF pattern were consistently least likely to be in the low BMI trajectory. Interestingly, compared with the Dutch reference group, nearly all feeding patterns of the Turkish/Moroccan group were associated with lower odds for the low trajectory. In line with our results, previous study [30] found no association between EBF during the first 6 months and BMI development in immigrant (non-Swedish) children, whereas EBF was associated with lower BMI trajectory in Swedish children. These findings suggest ethnic specific associations between infant feeding and growth. This could be explained by differences in other infant feeding practices between certain ethnic groups, for which we lacked information, such as feeding frequency, diet quality or bottle feeding practices [31, 58, 59]. Especially, macronutrient intake after the breastfeeding period may influence the association between breastfeeding and body fat [60]. Also other determinants, such as genetic predisposition [61] or maternal factors such as gestational weight gain or diet during pregnancy are suggested to be important for the ethnic differences in childhood overweight [62].

Our study has several strengths. These include the use of prospective data on infant feeding and growth in a multi-ethnic population. To our knowledge, we are the first to examine associations of infant feeding patterns, including milk feeding type and CF timing, with BMI trajectories across ethnic groups. Unlike some previous studies [9, 10], we modelled BMI trajectories using unstandardized BMI, which is shown to be better suited for longitudinal measures than z-scores [63]. Furthermore, we adjusted for several maternal and infant confounders. However, some limitations should be acknowledged. First, results are based on observational data and do not allow to make conclusions about causal effects of feeding on growth. Although our analyses were adjusted for birth weight, it cannot be ruled out that the observed BMI trajectories are the result of reverse causality. For instance, parents of children with low BMI early in life may respond to this by overfeeding the child (because the child’s growth is below the norm used by YHC, hence extra feeding is advised). In that case a high BMI later in life is not simply caused by feeding itself but the underlying cause of this trajectory may be a response to a low BMI (or weight for height) earlier. Second, our study population included two ethnic minority groups which limits the generalizability of our results to all populations. Also, we conducted ethnic specific analyses (planned a priori) in which some of the feeding groups of Turkish/Moroccan children were of limited sample size. Therefore, these results should be interpreted with caution. Fourth, although we adjusted our analyses for several known confounders, some of the continuous covariates were categorized in order to avoid estimating non-linear associations, which would have further increased the model complexity. However, categorization of the continuous covariates may be considered suboptimal [64]. Finally, we lacked information on other potentially important maternal or infant related factors, such as gestational weight gain or the quantity and quality of the diet during infancy, which may have also explained some of the observed associations. Future studies should therefore include more detailed nutritional information on infant feeding.

## Conclusions

Infant feeding patterns are associated with BMI trajectories during the first year of life with specific ethnic differences. Future studies should take the role of ethnicity into account in the associations between infant feeding and growth.

## Supplementary Information

**Additional file 1.**

## Data Availability

Data are available upon request due to ethical restrictions related to protecting patient confidentiality. Researchers who are interested in using data for research purposes can apply for access to the ABCD study data by contacting the research committee at abcd@amc.uva.nl or the principal investigator T. G. M. Vrijkotte (t.vrijkotte@amc.uva.nl).

## Abbreviations

CF: Complementary feeding
BMI: Body mass index
ABCD: Amsterdam Born Children and their Development study
EBF: Exclusive breastfeeding
YHC: Youth Health Care
LCMM: Latent class mixed effect model
