# Supplementary material for: Feeding patterns and BMI trajectories during infancy: a multi-ethnic, prospective birth cohort
Source: BMC Pediatr. 2021 Jan 13;21:34. doi: 10.1186/s12887-020-02456-4 (PMC7805191; doi:10.1186/s12887-020-02456-4)
Supplement: Supplementary file 1 — Additional file 1. [file 12887_2020_2456_MOESM1_ESM.docx]

**Table S1.** Associations of infant feeding patterns with BMI trajectories, sensitivity analysis based on the type of milk feeding provided during 0-6 months^1-3^

|  |  | BMI trajectory | | | | | |
| --- | --- | --- | --- | --- | --- | --- | --- |
|  |  | ‘’low’’ |  | ‘’mid-low’’ |  | ‘’mid-high’’ |  |
| % (n) |  | OR (95% CI) | p-value | OR (95% CI) | p-value | OR (95% CI) | p-value |
|  | Feeding pattern^4^ |  |  |  |  |  |  |
| 5.4 (192) | EBF/ early CF | 1.14 (0.57, 2.27) | 0.71 | 0.66 (0.26, 1.65) | 0.37 | 0.45 (0.21, 0.93) | 0.03 |
| 13.0 (459) | EBF/ late CF (reference) | - |  | - |  | - |  |
| 26.7 (942) | Mixed/ early CF | 1.71 (0.94, 3.10) | 0.08 | 2.35 (1.21, 4.58) | 0.01 | 0.73 (0.42, 1.28) | 0.27 |
| 35.2 (1240) | Mixed/ late CF | 2.45 (1.40, 4.27) | <0.01 | 2.17 (1.15, 4.11) | 0.02 | 0.91 (0.53, 1.58) | 0.74 |
| 11.5 (405) | Formula/ early CF | 0.87 (0.45, 1.70) | 0.69 | 1.09 (0.50, 2.36) | 0.84 | 0.32 (0.16, 0.62) | <0.01 |
| 8.1 (286) | Formula/ late CF | 1.34 (0.54, 3.31) | 0.53 | 2.35 (0.90, 6.10) | 0.08 | 0.61 (0.25, 1.47) | 0.27 |

^1^Values are OR based on LCMM. ^2^Reference BMI trajectory ‘’high’’. ^3^Adjusted for ethnicity, educational level, pre-pregnancy BMI, parity, smoking, sex, preterm birth, birth weight. ^4^feeding patterns are based on the type of milk feeding provided during 0-6 months period.

**Table S2.** Ethnic-specific associations of infant feeding patterns with BMI trajectories (model 2) (Dutch, Turkish/Moroccan), sensitivity analysis based on the type of milk feeding provided during 0-6 months^1-3^

|  |  | BMI trajectory | | | | | |
| --- | --- | --- | --- | --- | --- | --- | --- |
|  |  | ‘’low’’ |  | ‘’mid-low’’ |  | ‘’mid-high’’ |  |
| %^2^ (n) |  | OR (95% CI) | p-value | OR (95% CI) | p-value | OR (95% CI) | p-value |
|  | Feeding pattern^4^ |  |  |  |  |  |  |
|  | Dutch |  |  |  |  |  |  |
| 5.2 (150) | EBF/ early CF | 1.39 (0.57, 3.36) | 0.47 | 1.05 (0.34, 3.24) | 0.93 | 0.63 (0.22, 1.83) | 0.40 |
| 13.2 (380) | EBF/ late CF (reference) |  |  |  |  |  |  |
| 26.1 (752) | Mixed/ early CF | 2.12 (1.11, 4.07) | 0.02 | 3.03 (1.43, 6.45) | 0.00 | 0.86 (0.39, 1.90) | 0.71 |
| 34.7 (1000) | Mixed/ late CF | 2.69 (1.28, 5.65) | 0.01 | 2.67 (1.17, 6.08) | 0.02 | 1.03 (0.38, 2.81) | 0.95 |
| 12.1 (348) | Formula/ early CF | 0.99 (0.56, 1.76) | 0.97 | 1.32 (0.61, 2.85) | 0.49 | 0.37 (0.15, 0.89) | 0.03 |
| 8.7 (250) | Formula/ late CF | 1.42 (0.59, 3.45) | 0.44 | 2.68 (1.04, 6.87) | 0.04 | 0.68 (0.24, 1.88) | 0.46 |
|  | Turkish/Moroccan |  |  |  |  |  |  |
| 6.5 (42) | EBF/ early CF | 0.46 (0.16, 1.36) | 0.16 | 0.06 (0.00, 3.37) | 0.17 | 0.20 (0.05, 0.83) | 0.03 |
| 12.3 (79) | EBF/ late CF | 0.46 (0.12, 1.69) | 0.24 | 0.65 (0.18, 2.41) | 0.52 | 0.90 (0.30, 2.74) | 0.85 |
| 29.5 (190) | Mixed/ early CF | 0.27 (0.10, 0.69) | 0.01 | 0.78 (0.31, 1.97) | 0.59 | 0.51 (0.21, 1.21) | 0.13 |
| 37.3 (240) | Mixed/ late CF | 0.77 (0.35, 1.70) | 0.52 | 0.75 (0.30, 1.87) | 0.54 | 0.56 (0.23, 1.40) | 0.22 |
| 8.9 (57) | Formula/ early CF | 0.24 (0.07, 0.87) | 0.03 | 0.38 (0.10, 1.44) | 0.15 | 0.20 (0.05, 0.78) | 0.02 |
| 5.6 (36) | Formula/ late CF | 0.05 (0.00, 1.42) | 0.59 | 1.15 (0.23, 1.42) | 0.87 | 0.47 (0.10, 1.42) | 0.32 |

^2^The percentages for the different feeding patterns are within the Dutch or within the Turkish ethnicity. ^4^Feeding patterns are based on the type of milk feeding provided during 0-6 months period.
